# Interdisciplinary behavioral health provider perceptions of implementing the Collaborative Chronic Care Model: an i-PARIHS-guided qualitative study

**DOI:** 10.1186/s43058-023-00407-5

**Published:** 2023-03-30

**Authors:** Bo Kim, Jennifer L. Sullivan, Karen L. Drummond, Samantha L. Connolly, Christopher J. Miller, Kendra Weaver, Mark S. Bauer

**Affiliations:** 1 VA Boston Healthcare System, 150 South Huntington Avenue, Boston, MA 02130 USA; 2 Harvard Medical School, 25 Shattuck Street, Boston, MA 02115 USA; 3 VA Providence Healthcare System, 385 Niagara Street, Providence, RI 02907 USA; 4 Brown University School of Public Health, 121 South Main Street, Providence, RI 02903 USA; 5 Central Arkansas Veterans Healthcare System, 4300 West 7th Street, Little Rock, AR 72205 USA; 6 University of Arkansas for Medical Sciences, 4301 West Markham Street, Little Rock, AR 72205 USA; 7 VA Office of Mental Health and Suicide Prevention, 810 Vermont Avenue NW, Washington, DC 20420 USA

**Keywords:** Collaborative care, i-PARIHS framework, Mental health, Interdisciplinary care, Qualitative research, Collaborative Chronic Care Model (CCM)

## Abstract

**Background:**

The evidence-based Collaborative Chronic Care Model (CCM), developed to help structure care for chronic health conditions, comprises six elements: work role redesign, patient self-management support, provider decision support, clinical information systems, linkages to community resources, and organizational/leadership support. As the CCM is increasingly implemented in real-world settings, there is heightened interest in understanding specific influences upon implementation. Therefore, guided by the Integrated Promoting Action on Research Implementation in Health Services (i-PARIHS) framework, we (i) identified innovation-, recipient-, context-, and facilitation-related influences on CCM implementation and (ii) assessed the influences’ relationship to each CCM element’s implementation.

**Methods:**

Using semi-structured interviews, we examined interdisciplinary behavioral health providers’ experiences at nine VA medical centers that implemented the CCM. We used i-PARIHS constructs as a priori codes for directed content analysis, then analyzed the data for cross-coding by CCM element and i-PARIHS construct.

**Results:**

Participants (31 providers) perceived the CCM innovation as enabling comprehensive care but challenging to coordinate with existing structures/procedures. As recipients, participants recounted not always having the authority to design CCM-consistent care processes. They perceived local leadership support to be indispensable to implementation success and difficult to garner when CCM implementation distracted from other organizational priorities. They found implementation facilitation helpful for keeping implementation on track. We identified key themes at the intersection of i-PARIHS constructs and core CCM elements, including (i) the CCM being an innovation that offers a formal structure to stepping down care intensity for patients to encourage their self-management, (ii) recipients accessing their multidisciplinary colleagues’ expertise for provider decision support, (iii) relationships with external services in the community (e.g., homelessness programs) being a helpful context for providing comprehensive care, and (iv) facilitators helping to redesign specific interdisciplinary team member roles.

**Conclusions:**

Future CCM implementation would benefit from (i) facilitating strategic development of supportive maintenance plans for patients’ self-management, (ii) collocating multidisciplinary staff (on-site or virtually) to enhance provider decision support, (iii) keeping information on available community resources up to date, and (iv) making clearer the explicit CCM-consistent care processes that work roles can be designed around. This work can inform concrete tailoring of implementation efforts to focus on the more challenging CCM elements, which is crucial to better account for multiple influences that vary across diverse care settings in which the CCM is being implemented.

**Supplementary Information:**

The online version contains supplementary material available at 10.1186/s43058-023-00407-5.

Contributions to the literature

- We examined how implementing each core element of the evidence-based Collaborative Chronic Care Model (CCM) is influenced by various characteristics of the setting and individuals involved, attributes of the CCM, and strategies used to promote the CCM’s uptake.
- This work demonstrates a systematic process for devising specific implementation plans that target the CCM’s core elements that are more challenging to implement than others.
- Such element-specific plans are especially crucial when the CCM is being implemented in low-resource settings that call for careful prioritization in how available resources are allocated for implementing different CCM elements that are more or less feasible to focus on.

## Background

Numerous effectiveness trials have found the Collaborative Chronic Care Model (CCM) to be useful in structuring mental health care to be anticipatory, coordinated, and patient-centered [1–3]. The CCM consists of six core elements: work role redesign, patient self-management support, provider decision support, clinical information systems, linkages to community resources, and organizational/leadership support [4–6]. Table 1 describes and provides examples of each CCM element. These elements are meant to be flexibly implemented according to local needs, capabilities, and priorities [2]. Thus, as the CCM is being increasingly implemented in real-world settings, of heightened interest to the field is how implementation is influenced by characteristics of the setting, attributes of the CCM, and strategies used to promote the uptake of the CCM.

**Table 1:** Elements of the Collaborative Chronic Care Model (CCM), adapted from [7]

CCM element	Definition	Example
Work role redesign	Providing care that anticipates patients’ needs and preferences through redesigning processes within an interdisciplinary team structure	Establishing a care manager role to conduct phone-based assessments with patients, place reminder calls, and follow up after appointments to ensure continuity of care
Patient self-management support	Enhancing patients’ self-management skills to help them work toward wellness outside of treatment sessions	Supporting self-management and coping skills for patients to use between appointments (e.g., via homework and mobile apps)
Provider decision support	Ensuring that the treatment team’s providers have access to needed clinical expertise	Making available treatment manuals, medication algorithms, and streamlined access to specialty consultation (in cases where there is a concern outside of their area of expertise)
Clinical information systems	Using electronic/automated mechanisms to enhance evaluation and coordination of care, with an emphasis on caring for patient populations or panels	Establishing a registry or panel of patients for whom the treatment team is responsible, through which the team can track outcomes, including measurement-based care, across the whole team’s caseload to enable targeted feedback to providers
Linkages to community resources	Facilitated or systematic relationships with entities outside of the immediate treatment setting to support care delivery and community integration	Routinely using local community resources or web-based peer support services located outside of the clinical setting (e.g., recreational groups, housing assistance programs, transportation services, Alcoholics Anonymous, Depression and Bipolar Support Alliance)
Organizational/leadership support	Providing resources and support to the treatment teams from various levels within the organization, including executive level leaders as well as more direct line supervisors and managers in mental health specialty care services	Providing dedicated time to attend treatment team meetings and incentivizing attendance; ensuring that teams are fully staffed and have the support needed to enact the other CCM elements

Understanding these influences can enable CCM implementation efforts to be tailored to account for setting-specific needs, thereby enhancing the likelihood of successful CCM uptake within real-world settings. Successful implementation, according to the Integrated Promoting Action on Research Implementation in Health Services (i-PARIHS) framework, “results from the facilitation of an innovation with the intended recipients of implementation in their local, organizational, and health system contexts” [8]. Applied to CCM implementation, i-PARIHS provides an organizing framework through which to consider influences upon specific implementation efforts. Namely, the influences can be identified as belonging to one or more of i-PARIHS’ four constructs: (i) the *innovation* being implemented, (ii) the *recipients* of the implementation effort, (iii) the local, organizational, and health system *context* in which the implementation is carried out, and (iv) the *facilitation* of the implementation.

We recently conducted an i-PARIHS-guided randomized trial that examined the effectiveness of implementation facilitation in establishing the CCM within outpatient general mental health care clinics at nine United States Department of Veterans Affairs (VA) medical centers [9]. Implementation facilitation is a multifaceted strategy of interactive problem solving and support [10]; for our trial, implementation facilitation included a study-funded external facilitator who brought content and process improvement expertise to each implementation site and worked closely with a site-funded internal facilitator, who offered the knowledge of the site’s organizational culture and existing procedures [11]. Three study team members served as the external facilitator for three sites each, and the internal facilitator for each site was an employee who had at least some quality improvement experience and was identified by the mental health service director at the site. We chose i-PARIHS as the guiding framework because it specifically identifies facilitation as the “active ingredient” that leads to successful implementation [8]. The trial evaluation included a qualitative component in which we conducted semi-structured interviews with outpatient mental health providers at the nine sites. The interviews were focused on questions about the providers’ experiences with each of the CCM elements, and we have previously reported results from these interviews for the extent to which CCM elements were existent pre-implementation [12] and changed by the implementation effort [7]. Neither the CCM element-specific interview data nor the providers’ responses to additional questions about general enablers of and barriers to implementation, however, have yet been examined in terms of (i) innovation-, recipient-, context-, and facilitation-related influences on CCM implementation and (ii) how these four i-PARIHS constructs relate to each core CCM element. i-PARIHS guided our facilitation-based approach of implementing the CCM, and was part of our a priori plan for evaluating the implementation effort across the i-PARIHS constructs of innovation, recipient, context, and facilitation [11]. Without understanding the ways in which specific characteristics of these constructs relate to each core CCM element, it is difficult to plan future CCM implementation and sustainment efforts that are tailored to focus particularly on elements that were previously difficult to implement.

To fill this critical gap, we carried out a secondary qualitative analysis of provider interview data using i-PARIHS as the analytical framework to explore influences upon implementation. We sought to answer the following two research questions: (i) What are innovation-, recipient-, context-, and facilitation-related influences upon CCM implementation? (ii) How do these influences relate to the implementation of each core CCM element? In this paper, we describe our qualitative analytical approach, then share our findings regarding provider perceptions and experiences of CCM implementation as viewed through the i-PARIHS lens. We discuss implications of our findings for how future CCM implementation efforts can take innovation-, recipient-, context-, and facilitation-related influences into consideration, particularly for establishing care processes that align to specific core elements of the CCM. To this end, we include in our discussion a proposed four-step approach for core CCM element- and i-PARIHS construct-specific implementation planning.

## Methods

The study was reviewed and approved by the VA Central Institutional Review Board. Details regarding the procedures undertaken for the overall CCM implementation trial, which serves as the context for this study, have been previously published [9, 11]. Similarly, details regarding data collection for the semi-structured provider interviews have also been previously published [7]. We thus provide below only a brief overview of the steps that we took for data collection, then describe in further detail the steps that we took for conducting i-PARIHS-guided data analysis. Additional file 1 provides the Consolidated Criteria for Reporting Qualitative Research (COREQ) Checklist [13] that we consulted in reporting our work.

### Study population

We targeted all outpatient mental health providers who took part in the CCM implementation trial at the nine VA medical centers. Their disciplines included nursing, psychiatry, psychology, social work, and vocational rehabilitation. Per i-PARIHS, these providers (rather than their patients) were the recipients of CCM implementation, since their adoption of CCM (at the level of the outpatient mental health team) was the goal of implementation. We also included administrative support staff, since VA’s official guidance for staffing CCM teams considers administrative support staff as a part of the team, expecting them to play a critical role in ensuring that record/document management and other administrative team tasks are expertly covered. These administrative support staff are often the very first CCM team member who patients interact with when seeking care, and they are responsible for scheduling patients’ appointments and spearheading other important patient-facing tasks.

### Data collection

We recruited participants via email, sending up to three emails per potential participant. We gained access to email addresses of potential participants through mental health service leadership at each site, and the potential participants’ decisions whether to participate were not shared with their leadership. Each interview lasted between 30 and 60 min, and used a team-developed interview guide [7] focused on the extent to which the participant perceived elements of the CCM to have been implemented at their site. Interview questions also inquired about the participant’s experiences with the CCM and their perceived barriers and enablers influencing CCM implementation, as well as about whether they attributed care process changes to the implementation effort. Interviews were digitally recorded and professionally transcribed verbatim in all but two cases, in which per the participant’s preference, detailed notes were taken instead of recording. In each of these cases, to help ensure the accuracy of the data collected, there was a dedicated notetaker present in addition to the interviewer. Following the interview, the pair reviewed the notes together and drew on both of their recollections of the interview to make any needed edits to the notes. All the interviewers and notetakers were experienced qualitative researchers with expertise in proper and rigorous use of notes for interview-based studies [14, 15].

### Data analysis

Interview data were coded using a directed content analysis approach [16], utilizing an i-PARIHS-informed codebook [17]. We selected and refined codes from the codebook to group and eliminate sub-constructs that were difficult to distinguish from one another in this context and less directly relevant, respectively, regarding our specific CCM implementation effort.

Coding was conducted independently by KD and JS, and discrepancies were resolved through consensus discussions. Key themes based on the data, as well as their associated examples, were summarized into a structured template for each of the four constructs (Additional file 2). Specifically:

- BK, JS, and KD independently reviewed the data for the four constructs.
- JS and BK served as analysts for creating summaries for each construct, and each reviewed and discussed the other’s work. KD then reviewed revised summaries for all four constructs and met with JS and BK to discuss and finalize them.

We used the completed summaries for each construct to recapitulate our findings regarding the innovation-, recipient-, context-, and facilitation-related themes overall, which are presented in the Results section.

Data were also examined for cross-coding by CCM element (coding available from our previous work [7]) and i-PARIHS construct. We first identified element-construct pairs that were most prominent in the data (e.g., data relating to both the CCM element of work role redesign and the i-PARIHS construct of context), then generated descriptive summaries of themes from these cross-coded data (through independent reviews and consensus-reaching discussions by JS, BK, and KD, similar to the analytical process described above for generating construct-level summaries). We held discussions to agree on (i) which themes to report as being key, (ii) how to structure their reporting (e.g., first by i-PARIHS construct, then by prominent CCM elements per construct), and (iii) implications of our findings. We also reviewed the potential relevance of our findings to recommendations for CCM implementation that were put forth by our team’s previous publications [7, 12].

## Results

Participants included 31 interdisciplinary behavioral health providers from the nine VA medical centers that took part in the CCM implementation trial. The number of participants from each medical center ranged from one to five. As two of the nine sites had only one participant, assessment of within-site saturation was not feasible for those sites. We thus aggregated data across sites rather than analyzing each site separately. The disciplines represented by the participants included nursing (19%), psychiatry (23%), psychology (16%), social work (23%), and vocational rehabilitation (7%). The remaining 12% of the participants included peer support specialists, addiction counselors, pharmacists, and administrative support staff.

Table 2 provides examples of key themes that we found at the intersection of i-PARIHS constructs and CCM implementation, both for CCM implementation overall and for core CCM elements. Table 3 provides supporting quotes for i-PARIHS construct-related themes for CCM implementation overall. Further descriptions of our findings below are arranged by the i-PARIHS constructs of innovation, recipients, context, and facilitation (to address the first of our two research questions). In each case, we begin with overall findings related to the i-PARIHS constructs, before delving into CCM element-specific findings. We also specify which themes were more prominent for certain CCM elements than for others, where appropriate (our second research question).

**Table 2:** Examples of key themes at the intersection of Integrated Promoting Action on Research Implementation in Health Services (i-PARIHS) constructs and Collaborative Chronic Care Model (CCM) implementation, for CCM implementation overall and for core CCM elements

	**Innovation**	**Recipients**	**Context**	**Facilitation**
CCM implementation overall	• Empowers providers to better navigate the system to help meet patient needs • Needs more clarity around what care delivery processes embody CCM principles	• Are familiar with patient-centered and recovery-focused care • Do not always have the authority to design care processes	• Balancing implementation alongside concurrent change initiatives is challenging • CCM being a national recommendation serves as an impetus for local implementation	• External facilitators help set reasonable expectations and are responsive to inquiries • Internal facilitators’ experience with quality improvement and team/process skills is valuable
CCM element: work role redesign	• Better allows multidisciplinary input • Brings about more efficient workflow for referrals	• Leverage autonomy where possible to implement CCM • Grow together as a team and build trust in playing synergistic roles	• Frequent staff transitions are challenging • Existing adjacent site-specific processes may or may not fit with CCM	• External facilitator provides overall CCM knowledge • Internal facilitator leads redesign of specific team roles
CCM element: patient self-management support	• Better enables step-down in care intensity for patients • Makes an interdisciplinary team of providers available to patients	• Are aware of having group treatment plans • Explain to patients the new team-based mode of care delivery	• Provider experience with actively involving patients in their care is helpful • Use of evidence-based therapies that involve self-management support is helpful	• Internal facilitator discusses with team how to strategically plan supportive maintenance for medically improved patients
CCM element: provider decision support	• Enhances the frequency with which cases are discussed • Fosters a shared understanding of different expertise	• Consult team members from different disciplines • Consult expertise outside the team	• Collocation of providers helps CCM • Existing capability to assign additional signers to patient notes is used for CCM	n/a
CCM element: clinical information systems	• Is a useful reminder to more routinely deliver evidence-based care • Makes panel-based case management difficult without adequate information systems	n/a	n/a	• Both external and internal facilitator support increasing shared caseloads and creating shared panels across team
CCM element: linkages to community resources	n/a	• Share resources in team meetings and through pamphlets • Rely particularly on social work team members for knowledge	• Existing collections of available resources are helpful • Communicating effectively with community organizations is challenging	n/a
CCM element: organizational/leadership support	• Is met with resistance from leadership • Is not clear on the extent to which teams can design their own processes	• Use professional networks to solicit input from leadership • Need support to attend trainings to increase CCM-related skills	• Leadership does not always solicit input from providers in making CCM-related decisions • Leadership is not always aware of being non-inclusive	• Many sites had positive experiences with implementation facilitation • Isolated cases of mishandled communication or being from outside the service threatened team trust in facilitators

**Table 3:** Supporting quotes for Integrated Promoting Action on Research Implementation in Health Services (i-PARIHS) construct-related themes for Collaborative Chronic Care Model (CCM) implementation overall

**i-PARIHS construct**	**Themes (i-PARIHS sub-construct **[17]**)**	**Quotes**
**Innovation**	• CCM empowers providers to better navigate the system to help meet patient needs (relative advantage of the innovation)	“I think I know a little bit more how the other professions operate so I can kind of tell them how to prep the patient and maybe make some assumptions and really kind of give them more warm handoffs.” (Participant 804)
	• There is a need for more clarity around what care delivery processes embody CCM principles (usability of the innovation)	“I mean I think if there was a little more structure and guidance … maybe not policy but more procedure on how to do things, then people might feel a little more comfortable with it instead of just free floating, do whatever you want for 90 min [of team meeting] kind of thing.” (Participant 104)
**Recipients**	• Interdisciplinary collaboration and communication on the CCM team improved during the recipients’ participation in this project (collaboration and teamwork among recipients)	“… we kind of know each of our strengths and weaknesses, [are] working together so that we’re not having to assess the [same patient] five times, and reduce inefficiencies and play on all our strengths.” (Participant 804)
	• Changing the recipients’ practice norms was considered challenging (attitude of recipients toward the innovation)	“Unless their standard operating procedures are written, no, [they’re not] going to do it this way, they're going to do it the way they've always done it.” (Participant 101)
**Context**	• Engaging other clinical services for care process changes associated with implementation is challenging (networks and relationships influencing implementation)	“… in our clinic, primary care and mental health [services] just don’t get along really well. … we had [representatives] from primary care come [to] meet with us [and] work together but we still seem to hit some barriers and roadblocks with the integration of the two services.” (Participant 205)
	• CCM being a national recommendation serves as an impetus for local implementation (policies and priorities influencing implementation)	“… a recommendation that came out [from meeting with national leadership] … was that we were supposed to be in [CCM-based] teams and we were not.” (Participant 102)
**Facilitation**	• External facilitators help set reasonable expectations and are responsive to inquiries (planning/preparing for implementation)	“Our external facilitator came in and kind of gave us some more direction, kind of cleared a path for what we really need to be focusing on and I think now we have a better understanding and we’re more geared toward those goals.” (Participant 304)
	• Internal facilitators’ experience with quality improvement and team/process skills is valuable (using interpersonal skills to create a supportive environment)	“I really appreciated how [the team meeting] was run because I know having many people on a team, there’s different perspective, [so it] can also be difficult to keep things on track.” (Participant 912)

### Innovation

#### Innovation-related themes overall

Participants perceived the CCM as an innovation that enables providers to deliver more comprehensive care, although it can be challenging to coordinate with already existing structures/procedures and is mixed in terms of meeting patient preferences. They felt that the CCM empowers providers to better understand higher-level care delivery system workings, and in turn, better navigate the system to help meet patient needs.

To meet varying patient needs and to adapt to existing procedures, some participants voiced the desire for more flexibility in designing care processes that embody CCM principles. On the other hand, other participants suggested more clarity around what processes embody the CCM (and therefore should be worked on).

#### Innovation-related themes by CCM element

The above innovation-related influences on CCM implementation were perceived by the providers to be relevant to the implementation of specific core CCM elements.

##### Work role redesign

Implementing the innovation was viewed as better allowing multidisciplinary input on discussing clinical cases, and also as bringing about more efficient workflow for referrals. One participant remarked:

There’s such a flow now established to where I know exactly where [the referral] needs to go and who will see the person. (Participant 701)

##### Patient self-management support

Implementing the innovation was considered to better empower patients with self-management skills, enabling them to step down from a higher to a lower intensity of care when clinically appropriate, and also to better assure patients that the entire interdisciplinary team is available to provide support when needed. One participant noted:

… I think that is a positive thing for a patient to hear me [say] that -- okay, so this is [a] team who knows [you]. Not only one person but this team who knows [you] and if [I’m] not there, [there is] somebody else in that team who is already aware of what [you’re] going through. (Participant 105)

##### Provider decision support

Implementing the innovation was perceived to enhance the frequency with which cases are discussed among providers, and also to foster a shared understanding of the expertise across different disciplines that are available for providers to access. Frequent team discussions and understanding of team members’ strengths help with decision support within the team, by enabling team members to have ready access to and knowledge of each other’s expertise when they need such expertise to make informed clinical decisions. One participant mentioned*:*

I think that working together we were able to really understand what each team member actually could do, what’s in our tool bag. So we have an amazing group of members and we each have our own strengths. (Participant 805)

##### Clinical information systems

Implementing the innovation was seen as a useful reminder to more routinely incorporate evidence-based care into their work, and also as not yet making panel-based case management fully possible. One participant recommended:

I would say that for people who do this in the future, it is really important before they make any kind of move, for people to figure out data on -- in real life, how many patients does the clinic see, how many patients does each provider see, how are you going to do the teams before you do anything. (Participant 102)

Establishing such a concrete registry of the team’s patients is an important step towards tracking panel-wide trends and outcomes to guide the team’s care activities (e.g., to help decide which patients to focus team discussions and/or specialized treatment efforts on). Such tracking is a key example of enacting the CCM element of clinical information systems, as noted in Table 1.

##### Organizational/leadership support

Implementing the innovation was thought to have met with reluctance from leadership to rethink process changes already underway (even if rethinking could lead to better alignment to the CCM), and have also met with limited understanding by the providers regarding the extent to which teams can design their own processes. One participant expressed:

You know, I think part of it all was it was a bit of an inadvertent set-up from the beginning because of how some things were communicated from leadership to us. When this first started to get put in motion, it was presented as essentially the [CCM-based] team as our team and we can do whatever it is that we think we need to do to enhance services within the clinic. And so we started trying to get as creative as we could … with a vast majority of the ideas that we came up with, we were told to, at the beginning, do whatever you want and then we present something and then we were told no. (Participant 205)

### Recipients

#### Recipient-related themes overall

Staff mentioned being knowledgeable about several CCM principles, including patient self-management skills, consults and coordination, focusing on patient needs, and having a recovery focus. Team members gained skills to adopt CCM principles through using implementation materials (i.e., a CCM workbook [available upon request]). Staff at all sites mentioned that interdisciplinary collaboration and communication on the team had improved during their participation in this project.

Another theme that we found was not strictly team collaboration, but staff members identifying more as a team working together and being cohesive to better meet patient needs. There were three types of existing relevant professional networks the recipients took part in, including the network of staff within the CCM teams, the network outside the CCM team but within the medical center, and the community network outside the medical center and VA. Even with access to these networks, changing the recipients’ practice norms was considered challenging.

#### Recipient-related themes by CCM element

The above recipient-related influences on CCM implementation were perceived by the providers to be relevant to the implementation of specific core CCM elements.

##### Work role redesign

Recipients redesigning work processes to incorporate CCM elements into teams required those teams to grow together and build trust to collaborate and help span boundaries across disciplines through establishing synergistic roles. One participant commented:

I think our biggest focus has been on … our roles within [the CCM team] from each discipline and how we bring that together as a team. (Participant 510)

Also important was to leverage existing networks as well as staff power, authority, and autonomy to lead implementation efforts.

##### Patient self-management support

Team members mentioned that patient treatment plans were more coordinated because team members were aware of each other’s treatment approaches. One participant mentioned:

We would enhance [a patient’s treatment plan] as a team, or talk about something else that they might be able to work on or something else we might be able to add to their treatment progress. (Participant 305)

Overall patient self-management was driven more by patient needs. Some staff mentioned the need for more time to work with complex patients to discuss self-management or playing an active role in their own treatment plans. Sites also had to explain to patients that they were now being supported by a team versus an individual provider, which spanned existing networks/boundaries.

##### Provider decision support

Providers would seek support from their team members, as well as consulting other providers or experts as needed (in accessing community resources, utilizing supports outside of the team such as peer support, and/or managing co-existing health conditions or medications). These tasks required boundary spanning across roles within the team, and across services within the organization. In addition, obtaining decision support required teamwork and collaboration as well as leveraging existing networks to gather the necessary information to help make clinical decisions about patients.

##### Linkages to community resources

Staff at several sites mentioned that they were aware of clinicians on their teams with knowledge about community resources. This was especially the case around some disciplines such as social work having more information about resources. One participant remarked:

I had some ideas or thoughts on community resources [to offer to patients] because the social workers that I work with were well-versed in the community, …. (Participant 104)

Resources could be shared via word of mouth in team meetings or through pamphlets created within the team.

##### Organizational/leadership support

Recipients leveraged their existing networks to access or engage leaders in the work they were doing or changes they were requesting. They sought leadership support for hiring staff, allocating physical space to teams, changing productivity expectations, or attending trainings. If issues arose on teams spanning roles or silos in the organization, they involved a middle manager or supervisor to help overcome these challenges.

### Context

#### Context-related themes overall

Participants perceived context to be relevant to CCM implementation at the behavioral health service (local), medical center (organizational), and regional/national VA (health system) levels. At the behavioral health service level, support for implementation from clinic leadership was deemed essential, and participants felt that CCM-based teams helped foster the relationship between interdisciplinary providers. Along with needing more time and resources for CCM, staff mentioned two service management-related contextual factors that affected their ability to implement CCM — having control over their own schedules to fit in team meetings or CCM work, and having control over developing and implementing the team’s work processes themselves. At the medical center level, balancing implementation alongside other center-wide change initiatives, as well as engaging other clinical services for care process changes associated with implementation, were noted as challenges.

At the regional/national VA level, participants shared their views on how the CCM interacts with changing policies regarding community care eligibility for veterans, and how CCM implementation being a national recommendation served as an impetus for local implementation efforts.

#### Context-related themes by CCM element

The above context-related influences on CCM implementation were perceived by the providers to be relevant to the implementation of specific core CCM elements.

##### Work role redesign

The implementation context of prevalent staff transitions (retiring or otherwise leaving the clinic) was challenging, and the team’s role in the intake process was dependent on existing adjacent site-specific processes. One participant remarked:

We don’t have an intake clinic at our facility so … for the complex ones, we use our [CCM-based team] essentially as a way to take care of the general mental health needs for them so essentially doing that initial visit or intake … . (Participant 601)

##### Patient self-management support

The implementation context of providers already having experience with actively involving patients in their health care made it easier to continue such practice under the CCM, and experience with evidence-based therapies that explicitly involve self-management support was particularly pertinent.

##### Provider decision support

The implementation context of providers being collocated supported timely and frequent informal communication among them to collaborate on care delivery, and the existing capability to assign additional signers to patient notes in the electronic health record was further used under the CCM. One participant mentioned:

I can be put down as an additional signer on the note … which probably wasn’t a practice that we had before when we didn’t know who that other person would have been to put down an additional signer. (Participant 701)

##### Linkages to community resources

The implementation context of existing relationships with services outside VA (e.g., homelessness programs) was considered important to adequately provide comprehensive support for veterans. One participant shared their networking efforts:

… we … talked to different organizations … like a day care facility where [Veterans being treated within the team] could go and hang out with people like themselves and … have more interaction and bonding. (Participant 704)

##### Organizational/leadership support

The implementation context of leadership not soliciting input from providers and/or patients before requiring process changes was difficult to reconcile with the bottom-up design of processes encouraged by CCM implementation, and some leadership were not aware that their actions were not inclusive of frontline perspectives. One participant commented:

They [leadership] say I want to support this … so there might be a strong feeling by management that they support a change and they’ve made lots of good, great meaningful changes. … So I’m just concerned that they believe they are doing their part, they’re not aware of where they’re impeding progress. (Participant 504)

### Facilitation

#### Facilitation-related themes overall

Participants shared their experiences with both the external facilitator and the internal facilitator. External facilitators were viewed as knowledgeable, pleasant, encouraging, and responsive. Participants noted that external facilitators were helpful in guiding them through CCM implementation, suggested ways to move forward, set reasonable expectations, and learned team strengths and weaknesses. External facilitators also kept teams on course and were respectful of their time.

Regarding internal facilitators, participants referenced the importance of the internal facilitator’s role in directing teams between meetings via email, providing summaries, outlining action items, and tracking process outcomes (e.g., caseload). Internal facilitators’ experience with quality improvement and team/process skills was seen as valuable (or seen as an issue when the skills were not present).

#### Facilitation-related themes by CCM element

The above facilitation-related influences on CCM implementation were perceived by the providers to be relevant to the implementation of specific core CCM elements.

##### Work role redesign

The external facilitators would provide information about the CCM, and provide feedback on new processes the team would create (e.g., patient intake procedures) and suggestions for ways the team could improve those processes at the sites. The internal facilitators were more hands-on, attending more team meetings, writing up new processes for the team, and working with the team on defining specific team member roles. At one site, participants noted that an important part of the internal facilitator role was encouraging team meeting attendance, as without such encouragement the meetings could feature key staff absences. One quote representing this theme was:

[The] internal facilitator was really the broker in changing … the process … on how inpatient to outpatient care transitions occur. The internal facilitator was the, kind of the negotiating glue [in] the middle and had enough power to say, ‘Hey this is what we need to do to move this forward.’ (Participant 702)

##### Patient self-management support

There was only one quote where a team member mentioned having discussions with the internal facilitator about ways to strategically plan for supportive maintenance for patients especially when they may reach maximum medical improvement.

##### Clinical information systems

Two team members at one site mentioned working with both the external and the internal facilitator on increasing shared caseloads and creating shared panels across the team:

Part of the difficulty that we had was coming up with shared caseloads, like getting more people to be shared within our [team]. I know that we … switched over to this where they were consciously making these decisions and putting them on our [team] so that … we would have more shared caseloads … that was part of some of the discussions that the external facilitator was involved with … and that is something that the internal facilitator continues to look at. (Participant 907)

##### Organizational/leadership support

Staff described external facilitators as being cheerleaders and lending weight to the project for leadership negotiations. One staff member reported:

Our external facilitator was a really useful catalyst, was able to read and see our actions in a more appealing fashion to sell it to the leadership. (Participant 905)

Two sites had team members who described the external facilitator as playing a key role in bringing the importance of the project to the site and gaining leadership support. One team felt they lost some trust with an external facilitator regarding mishandling communication between the team and supervisors, which led to team members feeling confused. Although many sites experienced positive experiences with implementation facilitation, other staff members noted that having an internal facilitator from outside the mental health service could make it difficult to engage mental health leadership in supporting CCM-consistent care. In addition, an internal facilitator leaving the team resulted in:

… the team feeling rudderless because they know where we want to go but didn’t get much wind in our sails. The internal facilitator could energize the team and get it going in the right direction. (Participant 702)

## Discussion

Findings highlight that the CCM as an innovation empowers providers to help meet patient needs, yet needs more clarity around how care processes can be modified to embody CCM principles. The recipients of the CCM implementation — in this case, clinicians from several disciplines working together as part of a frontline mental health care team — were familiar with patient-centered and recovery-focused care approaches that the CCM encourages, yet did not always have the authority to redesign care processes to incorporate those approaches. The organizational context included many concurrent change initiatives, which made it challenging to prioritize CCM implementation. Partially counterbalancing this, an impetus for local implementation was the health system context of CCM-based care being recommended for outpatient general mental health services at all VA medical centers nationally. External facilitators were knowledgeable about CCM and were responsive to sites’ inquiries, and internal facilitators’ quality improvement expertise and team skills were deemed important for implementation.

Several of the identified themes at the intersection of i-PARIHS constructs and CCM elements are particularly worth noting in light of the field’s established and developing foci on related topics for CCM implementation. First, CCM team members viewed the CCM as an innovation that offers a formal structure to support stepping down care intensity for patients to encourage their self-management. As self-management is considered fundamental to recovery-oriented approaches to mental health care [18], promoting self-management is being increasingly considered a central responsibility of providers and health care systems [19]. Our finding suggests that CCM implementation may help providers and systems carry out this responsibility, in turn contributing to recovery-oriented care. To make this a reality, future CCM implementation would benefit from facilitating strategic development of supportive maintenance plans for patients’ self-management. Plans may be for symptom monitoring, relapse prevention, and coping strategies [20–23], and self-management apps and other technology-supported tools could better allow timely management [23, 24] that aligns to the CCM’s goal of delivering anticipatory, coordinated, and patient-centered care.

Second, we found that the recipients of CCM implementation (i.e., the CCM team members) were accessing their multidisciplinary colleagues’ expertise for provider decision support. Making informed clinical decisions is essential to minimize treatment delays and overtreatment, both of which can lead to inefficient use of care resources [25–27]. This is problematic now more than ever as health care costs continue to rise [28]. Given our finding that CCM implementation helps facilitate providers’ regular access to expertise and experience beyond their own, one example of this is to make available multidisciplinary staff (on-site or virtually) (e.g., teleintegrated care [29]). The resulting enhanced provider decision support can be expected to help promote the appropriate use of care resources and minimize incurring unnecessary health care costs. This expectation is supported by our related work that found our CCM implementation efforts to be associated with substantial cost savings [30], while still little is known about the influence specifically of improved CCM-facilitated provider decision support on health care costs.

Third, we found that relationships with external services in the community (e.g., homelessness programs) were considered to be a helpful context for providing comprehensive care. Services needed by individuals with mental illness are often not limited to mental health care and rather include a range of other health and social supports that are essential for their well-being [31–33]. These supports may include housing, employment, or legal services [34], which may be available within (e.g., [35, 36]) or outside of VA. Prior works have found care coordinators and case managers to play a central role in connecting individuals to these services outside of the health care system [37, 38]. Especially given the CCM’s goal of delivering coordinated care, future CCM implementation would benefit from keeping information on available community resources up to date, to which CCM team members can connect their patients. If a collection of resources is to be created, then it is important to specify the details of how the collection would be regularly updated and by whom, consistent with best practices for process standardization [39]. Such specification is also related to defining CCM team member roles, as noted below for the fourth theme discussed here.

Fourth, we found that facilitators helped redesign specific interdisciplinary team member roles. Even as interdisciplinary collaboration is being increasingly embraced as leading to better outcomes [40, 41], such collaboration is widely understood to be challenging [42]. Clarifying different disciplines’ respective roles is a particularly difficult aspect of interdisciplinary collaboration [43], and trainings and collaborative experiences of many health care professionals are only recently starting to be more interdisciplinary [44]. Furthermore, in the case of CCM specifically, the care model does not come with a set of pre-decided exact care processes to be implemented; the core CCM elements are principles to align to, rather than step-by-step guides to follow. Principles are then implemented based on specific local needs, resources, and priorities. Facilitators may thus often face the need to moderate discussions to establish not only role specifications but also care processes around which the roles would be specified. Previous works have described and/or assessed facilitator skills and characteristics that are important for implementation [45, 46], and i-PARIHS envisions the activities of the facilitator and the process of facilitation to be inherently flexible and responsive to what the recipients need to implement the innovation in their context [8]. However, as CCM implementation moves forward in multiple contexts (e.g., some without availability of a trained facilitator), future CCM implementation would benefit from making clearer (e.g., by clinic leadership with frontline input) the explicit CCM-consistent care processes that work roles can be designed around.

### Informing concrete planning of CCM implementation

Our previous analyses of the trial’s interview data identified CCM elements that were existent pre-implementation [12] and the extent to which they were changed by the implementation effort [7]. This follow-up study illuminates ways in which specific characteristics of the implementation’s innovation, recipients, context, and facilitation contribute to barriers and enablers influencing the implementation of each core CCM element. Taken together with our previous findings, this work can inform concrete planning of continued CCM implementation and sustainment efforts that are tailored to focus particularly on CCM elements that were found to be challenging to implement. Drawing additionally on i-PARIHS’ published guidance regarding construct-specific activities to focus on for implementation [8], the planning can be further honed to specify the activities that target the identified construct-specific influences on challenging CCM elements.

**Fig. 1:**
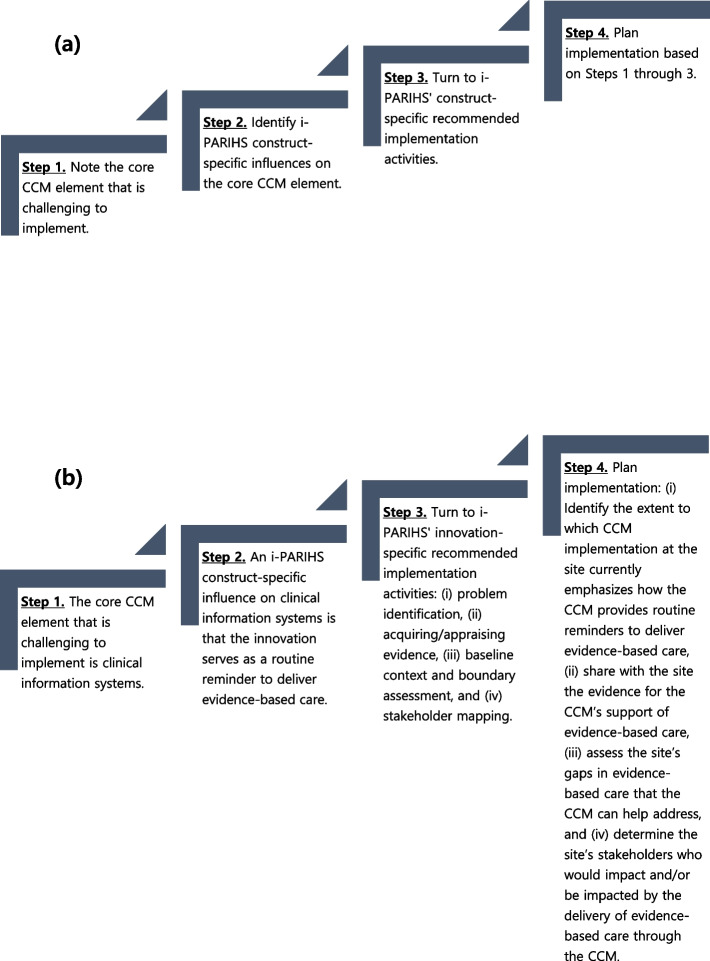
**a** A four-step approach for core Collaborative Chronic Care Model (CCM) element- and Integrated Promoting Action on Research Implementation in Health Services (i-PARIHS) construct-specific implementation planning, guided by [8]'s i-PARIHS construct-specific recommended activities. **b** An example of applying the four-step approach for core CCM element- and i-PARIHS construct-specific implementation planning

A four-step example of how this work enables such concrete planning is illustrated in Fig. 1. Figure 1a outlines the four steps, which are to Step 1, note the core CCM element that is challenging to implement; Step 2, identify i-PARIHS construct-specific influences on the core CCM element; Step 3, turn to i-PARIHS’ construct-specific recommended implementation activities; and Step 4, plan implementation based on Steps 1 through 3. Figure 1b shows an example of applying the four-step approach to implementation planning. Step 1, we note that we have previously found clinical information systems to be a CCM element that is neither prevalent at the sites pre-implementation nor notably established through our implementation effort that focused on all core CCM elements [7, 12]. Step 2, we confirm that the findings from this study (Table 2) identify *the innovation serving as a routine reminder to deliver evidence-based care* as an influence on the implementation of the CCM’s clinical information systems element. Step 3, we turn to i-PARIHS’ recommendations for innovation-related implementation activities [8], which are (i) problem identification, (ii) acquiring/appraising evidence, (iii) baseline context and boundary assessment, and (iv) stakeholder mapping. Step 4, based on Steps 1 through 3 (i.e., in planning for continued CCM implementation and sustainment efforts at a site that focus on enhancing the CCM’s clinical information systems element), we can devise activities that (i) identify the extent to which CCM implementation at the site currently emphasizes how the CCM provides routine reminders to deliver evidence-based care, (ii) share with the site available evidence for the CCM’s support of evidence-based care, (iii) assess the site’s gaps in evidence-based care that the CCM can help address, and (iv) determine the site’s stakeholders who would impact and/or be impacted by the delivery of evidence-based care through the CCM.

Importantly, the process illustrated in Fig. 1 is applicable to implementation efforts beyond those that are conducting i-PARIHS-guided CCM implementation. For any implementation effort that involves an innovation that is defined by core elements and is conceptually guided by a set of constructs relevant to implementation, the analytic approach that this study took can be applied to devise targeted implementation activities. These activities will ideally focus on one or more relevant constructs that are found to influence particular elements of the innovation that require more attention than others.

### Limitations

There are limitations to this work. First, the semi-structured interviews did not explicitly ask participants about each i-PARIHS construct. The interviews asked broader questions about participants’ experiences with the CCM and their perceived barriers and enablers influencing CCM implementation. This may have prevented gathering data from each participant about every i-PARIHS construct. However, this helped prevent the interview questions from leading the participants to consider constructs that otherwise would not have been on their minds. Second, this work is based on data from one multi-site CCM implementation trial that was conducted within the VA health care system. While further research is certainly needed to ascertain the applicability of both the analytic approach and the findings to CCM implementation in other contexts, it is encouraging that challenges to implementing CCM elements are not unique to VA [47, 48], and there thus exist opportunities for construct- and element-specific implementation planning to benefit CCM implementation efforts outside of VA as well. Third, our proposed four-step approach for core CCM element- and i-PARIHS construct-specific implementation planning has not yet been tested. Our study team currently has underway a subsequent funded i-PARIHS-guided CCM implementation trial that is informed by the extent to which our previous CCM implementation sites have been successfully sustaining the core CCM elements within their interdisciplinary mental health care delivery. Our explicit incorporation of construct-specific i-PARIHS recommendations in planning the new trial’s implementation will provide a strong opportunity to examine the utility of construct- and element-specific implementation activities. Fourth, there were two interviews for which, by participant preference, detailed notes were taken rather than audio-recording. For these interviews, we helped ensure data accuracy by utilizing a designated notetaker separate from the interviewer, as well as having the interviewer and the notetaker collaboratively finalize the notes through reflecting on both of their recollections. Importantly, all the interviewers and notetakers for the study were experienced qualitative researchers well versed in appropriate notetaking practices for interview-based studies [14, 15].

## Conclusions

As the evidence-based CCM is increasingly implemented across diverse care settings, there are likely multiple ways in which components of the innovation, recipients, context, and facilitation, as defined by i-PARIHS, influence the implementation of one or more of the CCM’s core elements. Through co-analyzing the experiences and perceptions of CCM implementation by both i-PARIHS’ established constructs and the CCM’s core elements, this work enables examination of how the different constructs influence the implementation of each CCM element. In turn, it makes possible a systematic process by which to devise i-PARIHS-guided construct-specific implementation plans that target the CCM’s core elements that are more challenging to implement than others. The context-informed specificity of such plans is especially crucial as CCM is implemented in low-resource settings and for those without equitable access to mental health care, so that planned activities can methodically account for what is more or less feasible within a particular implementation context.

## Supplementary Information

**Additional file 1.** COREQ (COnsolidated criteria for REporting Qualitative research) Checklist.

**Additional file 2.** Site i-PARIHS Summary.

## Data Availability

The data analyzed during the current study are not publicly available because participant privacy could be compromised.

## Abbreviations

CCM: Collaborative Chronic Care Model
COREQ: Consolidated Criteria for Reporting Qualitative Research
i-PARIHS: Integrated Promoting Action on Research Implementation in Health Services
VA: United States Department of Veterans Affairs
