# Supplementary material for: Interdisciplinary behavioral health provider perceptions of implementing the Collaborative Chronic Care Model: an i-PARIHS-guided qualitative study
Source: Implement Sci Commun. 2023 Mar 30;4:35. doi: 10.1186/s43058-023-00407-5 (PMC10061893; doi:10.1186/s43058-023-00407-5)
Supplement: Supplementary file 2 — Additional file 2. Site i-PARIHS Summary. [file 43058_2023_407_MOESM2_ESM.docx]

**Site i-PARIHS Summary**

**Template**

[Based on Ritchie MJ, Drummond KL, Smith BN, Sullivan JL, Landes SJ. Development of a qualitative data analysis codebook informed by the i-PARIHS framework. Implement Sci Commun. 2022;3(1):98.]

**Site:**

**Participant IDs/roles:**

*Instructions:*

*After each item/bullet point, indicate participant ID (e.g., [102]). Also after each one, label with [B] to indicate barriers or [F] to indicate facilitators as appropriate.*

*Example:*

- Providers do not have sufficient time to collect measures during the appointment [102] [B]

**Innovation**

Evidence/Underlying knowledge source for the innovation, including a) research/guidelines, b) clinical experience, c) patient needs/preferences/experiences, and d) local practice information showing innovation outcomes

Clarity or degree of understanding of what needs to be implemented

Complexity of the innovation

Degree of fit (or compatibility) between the innovation and the contextual norms/values/practices/operations

Degree of novelty of the innovation

Usability (or ease) with which the innovation can be used

Relative advantage (perceived or objective) of using the innovation

Trialability (or testing) the innovation

Observable results of the innovation

**Recipients**

Personal attributes of recipients

Skills and knowledge of recipients

How time, resources, and support affect recipients

Collaboration and teamwork among recipients

How existing networks affect recipients

Power, authority, and autonomy of recipients to direct or influence the actions of themselves and/or others

Presence of boundaries between recipients

General attitude of recipients toward the innovation

**Context**

*Note: For entries under any of the context characteristics below, also note whether they pertain to a) inner local context, b) inner organizational context, c) regional organizational context, d) national organizational context, or e) outer context.*

Leadership support for innovation implementation

Culture and climate of the context

History of innovation and change in the context

Policies and priorities (as well as policy drivers and mandates) that may influence implementation

Structures and systems within the context

Incentives and rewards that encourage or discourage innovation implementation

Infrastructure, resources, and support for innovation implementation

Evaluation, monitoring, and feedback processes conducted

Networks and relationships that can influence implementation

Political factors and dynamics that may influence implementation

Absorptive capacity of the context

**Facilitation**

Providing education/information to stakeholders

Collecting data and providing feedback to stakeholders

Building relationships, teams, and networks to support implementation

Enabling/Fostering change in the organization

Identifying problems and generating/selecting solutions

Planning/Preparing for implementation

Helping to identify, define, and fill stakeholder roles

Providing administrative and technical implementation support

Using interpersonal skills to create a supportive environment

Obtaining/Disseminating innovation or facilitation knowledge
